# Supplementary material for: Young adults’ experiences of using a young person’s mental health peer support app: A qualitative interview study
Source: PLOS Digit Health. 2024 Jul 31;3(7):e0000556. doi: 10.1371/journal.pdig.0000556 (PMC11290682; doi:10.1371/journal.pdig.0000556)
Supplement: S1 File — (DOCX) [file pdig.0000556.s001.docx]

## WP6 Interviews Topic Guide

1. **Experience of using Tellmi**

We’re going to start by talking about how you’ve found using the tellmi app

Broad opening question- can you tell me about your experience of using tellmi?

*Probes to use if participant is struggling to answer*

- - What areas of the app have you used (and what did you think about them)
    - What are your favourite parts of the app (and why)
  - How often do you use tellmi
  - Where are you or what are you doing when you usually use tellmi
  - What do you think about Tellmi compared to other mental health support
  - What do you think about the resources within tellmi (e.g., podcasts, user stories, art, educational resources, links to helplines)
- Can you tell me what you thought about the appropriateness of tellmi for your age group?
  - What do you think of the relevance or appropriateness of the content you see
- Can you tell me about your experience of personalizing the feed (e.g., filtering topics)

1. **Experience of peer support**

One of the main functions of the app is peer support, so we’re now going to spend some time talking about whether you used that and how you found it.

- Broad opening question- can you tell me about your experience of the peer support within tellmi?
- *Probes to use if participant is struggling to answer*
  - How often do you post and reply to posts
  - How do you feel about the amount of peer support available through the app (e.g., the number of replies that posts get)
  - How does peer support compare to other areas of app
  - What do you think of the format of peer support being posts and replies?
- In tellmi users typically see posts from people that are up to 2 years younger and 2 years older than them. How do you feel the peer support works for your age group?
  - What do you think about the appropriateness or relevance of the posts that you see?

*Benefits of peer support*

- Can you think of any benefits that peer support has had for you?
- *Probes to use if participant is struggling to answer*
  - Any specific benefits on e.g., mood, feelings, thoughts, social life

*Challenges of peer support*

- Can you think of any challenges of peer support that you experience?
- *Probes to use if participant is struggling to answer*
  - Any unhelpful parts, any negative impact, anything you don’t like about it
  - Anything that could be better about it

1. **Addressing outcomes of importance**

It would be helpful to get a sense from you of whether you feel that using the tellmi app had any impact on your wellbeing

- First establish what outcomes are meaningful for them, how they would want to feel after using the app, etc
  - When you first downloaded tellmi what were your hopes or expectations for the app
  - How did you want to feel after using tellmi
  - how does your experience of tellmi compare to what you hoped it would be
- Then establish whether tellmi helped with these

*Probes to use if participant is struggling to answer*

- - Has tellmi had any impact on your wellbeing or your life in any way?
    - Do you think it's had any positive impact for you?
    - Do you think it’s had any negative impact for you?
    - Has it had an impact for you on any specific areas (e.g., thoughts, feelings, mood, social life, school life) if so – what is the impact
  - Can you think of any ways that your wellbeing could be better supported through the app?

1. **App design**

Lastly, we want to explore how you found navigating and using the features within the app.

- What did you think of the design?

*Probes to use if participant is struggling to answer*

- - The artwork
  - The colours
  - Compared to other apps/support?
- How did you find interacting with the app?
  - Ease/enjoyment of use
  - Specific features
  - Compared to other apps/support?
- What was your experience of the sign up process?

1. **Anything else**

Any other feedback/comments/thoughts not yet covered
