# Supplementary material for: Young adults’ experiences of using a young person’s mental health peer support app: A qualitative interview study
Source: PLOS Digit Health. 2024 Jul 31;3(7):e0000556. doi: 10.1371/journal.pdig.0000556 (PMC11290682; doi:10.1371/journal.pdig.0000556)
Supplement: S2 File — (DOCX) [file pdig.0000556.s002.docx]

Evaluation of the Tellmi app – Distress protocol v1

If a participant indicates they are experiencing distress, or it is apparent to the researcher that the participant is experiencing distress (e.g., crying) the following steps will take place:

1. The interview will be paused and the participant will be asked how they are feeling. If they are experiencing severe distress, the researcher will recommend that the participant contacts mental health support or a loved one. The participant will be provided with a detailed and exhaustive list of resources and encouraged to contact them, the researcher may support them in this process if the participant would like.
2. If the participant feels ok and wishes to continue the interview, they will be reminded that they do not have to answer any questions that they find uncomfortable or upsetting, and also that they are free to withdraw from the interview at any time.
3. Following completion of the interview (whether the participant decides to resume or not), the participant will be sent a debrief form containing the detailed and exhaustive list of support services/ resources. They will also be reminded to contact the research team if they have any questions or wish to discuss their participation in the interview (including to withdraw their data within two weeks of the interview).

To help make sure the interviews are safe and comfortable spaces for participants, the following measures will be taken:

- The researcher will ensure that they leave time after interview slots in case the interview needs to be paused or the participant needs more time.
- The researcher will have the list of recommended support to hand should they need to discuss this with the participant during the interview.
- The researcher will spend time at the beginning reiterating the aims of the interview and the topics that questions may cover. They will then check with the participant whether they are comfortable to proceed.
