# Supplementary material for: Young adults’ experiences of using a young person’s mental health peer support app: A qualitative interview study
Source: PLOS Digit Health. 2024 Jul 31;3(7):e0000556. doi: 10.1371/journal.pdig.0000556 (PMC11290682; doi:10.1371/journal.pdig.0000556)
Supplement: Attachment — Submitted filename: Response to reviewers.docx [file pdig.0000556.s003.docx]

Reviewer #1: The authors conducted a qualitative study on users’ experiences of using a peer support mental health app. I appreciated the conciseness of the qualitative reporting, the language used, and the richness the themes generated. I consider the study quite merited.

Response: thank you for your kind and helpful feedback.

1. Evaluating the study reach is a considerable matter for the recruitment. In the Recruitment section, could consider describing to how many users the recruitment ad was shown to; in what time period; and how many clicked on the ad? You also mention that the study had a relatively small population to recruit from. How big was the population of users who had the capability to seeing the ad? How many of them were shown the ad?

Response: as the study ads were placed in the feed, it is unfortunately impossible to say with any confidence how many app users saw it or clicked on it. However, we do know that roughly 6,000 people use the app monthly and therefore may have seen the ad, although we cannot guarantee this. The study ads were shown for around 8 weeks (July – August 2023). This information has now been added to page 7 line 148:

‘Recruitment occurred via convenience sampling within the Tellmi app where the study was advertised within the app feed between July – August 2023.’

‘We do not know how many app users saw the advert, but we do know that roughly 6,000 people use the app monthly and therefore may have seen it. As the advert was programmed to appear as a post when scrolling within the primary feed, users may not have accessed that part of the app or scrolled far enough to see it, which may have posed barriers to recruitment.’

2. Regarding ethical approval, consider including the university name.

Response: the name ‘University of Bristol’ has now been added.

3. Please briefly characterize the topic guide used in the interview also in the main text. Also, do mention who conducted the interviews.

Response: Thank you for these suggestions. Both of these points have now been addressed on page 9, line 189-194:

‘Example questions included ‘can you tell me about your experience of using Tellmi’, ‘can you tell me about your experience of the peer support within Tellmi’, ‘can you think of any benefits/challenges of peer support from your experience’, ‘can you tell me whether Tellmi had any impact on your wellbeing’, and ‘what did you think of the design of Tellmi’. All interviews were conducted by a researcher (BC) with experience of qualitative research with young people on sensitive topics.’

4. What role, if any, did the Tellmi-platform developers and founders have in planning, conducting and analyzing the data?

Response: This role that Tellmi had in the research has now been clarified on page 7 line 142-145:

‘Tellmi played no role in data collection or analysis, but they were involved in facilitating recruitment. Additionally, Tellmi staff helped to design the topic guide, to ensure our data collection was informed by technical expertise.’

5. Understanding the design studied is highly important in the evaluation of human factors. In other words, the participants’ responses may be considerably influenced by the design. Therefore, please elaborate on features of the Tellmi-application in a dedicated section: What are its key features? Is it moderated? Is it free to use? Who is it for? How long has it been in operation? How many monthly active users does it have? Who has developed it? What previous studies have been conducted on it? What kind of instructions does it have for posting and responding? How is the design improved and how frequently? How does it compare to other services? What is it relationship with other services?

Response: Thank you for noting that more information was required regarding the tellmi app. A section has now been added to the methods (page 6-7, line 122-134):

‘Tellmi (previously MeeToo) was developed in 2017 and is a free to use UK-based digital peer support app in which young people (aged 11-25) can anonymously discuss a range of topics covering mental health and wellbeing with other age-banded peers. Users can post and respond to others’ posts within the primary feed and they can also access a directory of over 600 different wellbeing resources that includes signposting to various mental health charities and organisations, websites, helplines, etc. All posts and replies are pre-moderated for safety. Please see https://www.tellmi.help/ for more information. Tellmi engage in research activities involving their user-base to improve app design and extend its offering, for instance recent work to develop provision for high-risk users and to improve signposting within the app (16). It has been commissioned by the NHS, and a previous evaluation of Tellmi with younger children found that it helps young people to feel less alone and has a positive impact on their mental health (15).’

Information regarding monthly users has been added to page 8, line 163-164:

‘roughly 6,000 people use the app monthly’

6. Add the research questions at the end of the introduction in a dedicated section.

Response: The research questions have now been added as suggested on page 6 line 117-120:

‘Specific research questions were:

1)	What are young people’s experiences of using Tellmi?

2)	What are young people’s experiences of digital peer support?

3)	What meaningful impact does Tellmi have on young people’s wellbeing?

7. The Discussion explains that the study engaged in user-centered design by reporting the findings to Tellmi. This presents an intriguing option for future studies and iterations. As a reader, I would appreciate reading more about this considering that the ethos of this study is to create services (apps) that serve the users the best. How did this study further this aim, besides providing insights? How to foster impactful translation of research from findings to design iterations?

Response: thank you for raising this important point. Further information has been added to page 24 line 503-513:

‘This study was part of a broader academic-industry collaborative project, and a key strength of this research is the impact from feeding the findings back to Tellmi; findings were presented to Tellmi with recommendations for changes/additions that could improve the experience of peer support within the platform. This included specific design changes not covered in this article, as well as more conceptual ones such as providing more training for peers and considering the possibility of supervision for those taking more of an active role in the community. This builds upon other work we have done highlighting key elements of digital peer support that can help to extend its offering to those experiencing suicidal thoughts (16). It is also the aim that key principles from this paper could be applied to other digital peer support platforms.’

8. I do appreciate how the themes were structured and reported. However, would a brief table at the beginning of the results section help the reader keep them in mind?

Response: thank you for this suggestion. A table listing the themes has now been added on page 11 line 235.

9. “sospecialist” appears to be a misspelling.

Response: thank you for identifying this, this has now been corrected to ‘so specialist’

10. The study reports sampling as a limitation. This is also associated with the interview method used in the study – interviews appeal to certain subpopulation. What other methods could / should be used to explore the topic in the future?

Response: thank you for identifying this. This has now been addressed in the limitations on page 25 line 528-531:

‘Whilst we perceive a strength of this study to be the varying formats of interviews offered, it may still be that some users may have reservations about taking part in interview-based research. Anonymous surveys within the app may be appropriate for these individuals and should be considered for future research.’

11. At the end of discussion, the word co-design is used. However, there is some literature to suggest that the users should rather have the role of an informants than co-designer. I would advice rewording, while retaining the thought that evaluating the intervention with the users is important.

Response: thank you for raising this interesting point. This has now been reworded to reflect this on page 26 line 554-556:

‘Developing training and supervision informed by peers would be important for ensuring it meets their needs and is implemented appropriately.’

This has also been edited on page 27 line 570-572:

‘For example, how to create familiar environments within virtual reality based peer support may be an important consideration as this evolves (1). Again, involving young people in informing this will be essential here.’

This has also been edited in the author summary, page 3 line 53:

‘consulting young people to make sure the space feels friendly.’

Reviewer #2: This paper presents a timely and insightful exploration of young adults' experiences with a digital peer support smartphone app for mental health. The study's qualitative approach, utilizing reflexive thematic analysis, offers a good understanding of participants' perspectives and sheds light on the benefits and limitations of digital peer support in this demographic group. The identification of five key themes provides a structured framework for interpreting the findings, and the discussion offers valuable insights into the potential implications for enhancing the effectiveness of such interventions. However, further clarification on methodological details, validity, and reliability measures would strengthen the paper's rigor.

Response: Thank you for your kind and helpful feedback.

1. The authors mentioned usage time - a couple of months or weeks - but they don't display data regarding daily frequency - every day, several times a day, all the time... This may have an impact on the results. Looking at the interview guide there is a lot of information that is left out of their analysis, for example, usage data. In addition, the psychosocial state of young people who use the application is also possibly different in terms of stress, anxiety, depression, psychological well-being, and their responses will certainly be different.

Response: Information has been added on page 11 line 227-229 regarding frequency of use:

‘Four participants reported using Tellmi at least once a day, three used it at least once a week, whilst the remaining four participants gave no specific answer regarding frequency of use.’

Unfortunately, we don’t have data more specific than that for the frequency or length of use. Similarly, we did not collect data regarding any mental health conditions or psychosocial states of participants. This has been noted as a limitation on page 25 line 532-536:

‘Finally, no data were formally collected regarding participants’ length or frequency of Tellmi use. Similarly, no data were collected regarding participants current wellbeing or any mental health conditions they may have had. This information may have influenced the findings, so having an understanding of this would provide helpful context for this study. Future research should consider collecting this data where appropriate.’

2. The chat interview seems to be quite different in the type of information it collects, in the same way that a telephone call does not capture important non-verbal information. Nevertheless, some authors pointed that the use of online chatting for interviews is acceptable when video or online interviews aren't feasible; It's also suitable as an additional method if it complements the research and allows for diverse data sources (eg. <https://www.ncbi.nlm.nih.gov/pmc/articles/PMC10401366/>).

Response: thank you for this helpful insight. In this instance an audio/video interview wasn’t possible for this participant and so we agree that it was a suitable method. We have now included the reference you have provided here to support this on page 7 line 138-142:

“Whilst the data gathered from a chat-based interview as opposed to audio/video may be different, it is important to ensure the interviews are accessible to all who wish to take part. It has been identified that interviews using this format are acceptable and do not elicit data of a worse quality (2,3).”

3. The use of reflexive thematic analysis is appropriate for qualitative data, but it would be helpful to understand how rigor was maintained throughout the analysis process. Were there any efforts to mitigate potential bias during the analysis, such as reflexivity or member checking? What steps were taken to ensure the validity and reliability of the findings? For instance, were any strategies employed to enhance the trustworthiness of the data, such as triangulation or prolonged engagement with participants?

Response: thank you for this important suggestion. Reflexivity was practiced throughout the data collection and analysis process, and more information has been added now to page 9 line 196-198 to elaborate on this:

‘After each interview, BC wrote field notes documenting her experiences of conducting the interview, including the dynamic with the participant, any noteworthy observations and emotional reactions to the interview.

Data analysis

A third party company transcribed the interviews and the research team used reflexive thematic analysis to analyse the data (21,22). During analysis, BC continually reflected on her own positionality, acknowledging researcher subjectivity as integral to the process (23). Firstly, BC read all transcripts to become re-familiarised with the data as it was transcribed by a third party. BC then coded all transcripts using the comments function on Microsoft Word, coding both semantically and latently, and inductively and deductively. The corresponding field notes were considered alongside each transcript to provide context and enhance understanding of the data (24). To explore any further insights ZH independently coded two transcripts. BC compared the codes and found considerable overlap, with interpretations not previously considered being factored in to analysis. Codes were printed out and manually organised into themes in an iterative process where different organisations were considered until the final themes were settled on. This was discussed with LB and MJL throughout this process until the team were happy that the themes accurately represented the data. The transcripts were regularly checked to ensure the themes remained grounded in the data. During theme development, the research questions were held in mind, but this was flexible to allow unexpected findings to still be represented within the themes. For example, whilst we anticipated participants discussing the possibility of receiving unhelpful advice, we did not necessarily expect discussions around the fear of causing harm to others, but this felt like an important finding to report.’

4. The identification of five key themes is informative, but could the authors elaborate on how these themes were derived from the data? Did any unexpected findings emerge during the analysis?

Response: please see response to the above question.

5. In discussing the limits of digital peer support, could the authors provide more insight into why participants perceived the app primarily as a space for venting rather than for deeper exploration of mental health issues? I think a paragraph explaining/describing the Tellmi app is needed at the end of the introduction or in the methods, since this app has several resources available.

Response: thank you for this suggestion. A paragraph explaining Tellmi has been added on page 6-7 line 123-134:

‘Tellmi (previously MeeToo) was developed in 2017 and is a free to use UK-based digital peer support app in which young people (aged 11-25) can anonymously discuss a range of topics covering mental health and wellbeing with other age-banded peers. Users can post and respond to others’ posts within the primary feed and they can also access a directory of oer 600 different wellbeing resources that includes signposting to various mental health charities and organisations, websites, helplines, etc. All posts and replies are pre-moderated for safety. Please see https://www.tellmi.help/ for more information. Tellmi engage in research activities involving their user-base to improve app design and extend its offering. For instance, recent work to develop provision for high-risk users and to improve signposting within the app (16). It has been commissioned by the NHS, and a previous evaluation of Tellmi with younger children found that it helps young people to feel less alone and has a positive impact on their mental health (15).’

We believe that the participants primarily perceived the app as space for venting due to the lack of depth and person-centred support offered by peers, finding that they offer more comfort and reassurance than helping the poster to get to the root of and resolve issues. Please see page 21-22 line 448-454 for clarification:

‘It seemed as though the main function of digital peer support was a space for venting and releasing thoughts/feelings, rather than to necessarily resolve issues as it is not very person-centred or in-depth and peers were thought to primarily offer comfort rather than solutions. Consequently, the valuable role that professionals can play was also discussed by participants who felt that professionals could input in ways that peers cannot, for example providing advice that is evidence-based and better able to get to the root of problems.’

6. The suggestion to provide training and supervision to peers is interesting. Can the authors expand on what this training might entail and how it could be implemented effectively within the app? Are there any potential drawbacks or challenges associated with providing such training, such as resource constraints or concerns about over-professionalization? Consider potential power imbalances in the collaboration process between these profiles.

Response: thank you for this great point. More information has now been added on page 25-26 line 545-556 regarding possible options for training as well as the possible drawbacks:

‘As mentioned above, the #chatsafe campaign is a great example of how training could be delivered within an app through short videos, animations and text which coach the young person on how to respond to people in distress whilst keeping safe themselves. This has been evidenced to increase confidence and self-efficacy when communicating with peers in distress (32). However, this must be carefully considered as other research into digital peer support training has found that providing too much information in a short space of time may in fact decrease people’s readiness to support peers (34). Further, if some peers are trained and some are not, this may lead to a power imbalance within the app in which those who are not trained may lack the confidence to engage compared to their peers. Developing training and supervision informed by peers would be important for ensuring it meets their needs and is implemented appropriately.’

7. What are some potential avenues for future research based on the findings of this study? Are there any unanswered questions or areas requiring further exploration? How might the insights gained from this study inform the design and development of future digital peer support interventions for young adults? Maybe the authors can state more clearly some potential recommendations for future design and implementation of digital peer support for youth mental health based on results from their work.

Response: thank you for suggesting this. Ideas for future research have now been embedded within the discussion, for example page 22 line 456-461:

‘There may be individual differences here in that, whilst difficulties accessing services remain a persistent and pervasive issue, peer support may provide comfort, normalisation of experiences, relief and reassurance (25), but for some this is not enough and so specialist support may be required. Future research could help to untangle this relationship by identifying who may benefit most from peer support and who may need further support.’

And page 23 line 490-500:

Research has identified burnout and the stress of the role as challenges to youth peer support in face-to-face settings, with supervision and boundaries recommended to protect against this (33). However, these principles may not directly translate to digital peer support where there may be no supervision in place and limited guidance around boundaries. Future research should explore the impact of the length of engaging with digital peer support on peers’ wellbeing and perception of their role and responsibility. There may be some value in considering restrictions around how many posts peers can reply to as a way of introducing boundaries and reducing this pressure. Artificial intelligence could possibly be employed here to monitor users’ frequency of replies and offer supportive strategies to help prevent peer burnout, and exploring this in future studies may prove insightful.’

With regards to the insights informing design and development of digital peer support, the implications and recommendations have now hopefully been clarified, for example page 26 line 564-567:

‘Finally, digital peer support spaces presenting as informal and familiar was perceived as comforting and accessible for young people. This should be taken into consideration when designing digital peer support for young people to mimic platforms that are accessible to young people, such as social media.’

And page 26 line 557-563:

‘In addition to providing training and supervision, digital peer support platforms should emphasise the importance of boundaries and devise ways of constructing these within apps. One recommendation is exploring limits on how many posts an app user can reply to within a certain time frame may help to ease some of the pressure. This is an important consideration when designing digital peer support to ensure that all users are kept safe.’

8. Overall, despite the small sample, this paper contributes valuable knowledge to the field of digital mental health interventions and invites further inquiry into optimizing peer support approaches for young adults.

Response: thank you for your helpful review.

Reviewer #3: Peer review April 26 2024 Young adults’ experiences of using a young person’s mental health peer support app:
a qualitative interview study

Thank you to the authors for this interesting contribution to the literature. The authors write an article about the use of digital peer support methods such as mobile applications in the context of youth with mental health conditions. They authors used an interview approach and a qualitative interview which is an appropriate and important methodology to probe deeper into the subjective experiences of the users of these platforms. It appears users find certain benefits of using peer support apps that fill a gap between access to care and finding someone who is more relatable to them (ie. a peer and not an authority figure). I believe this is a valuable finding and this paper highlights certains strengths and weaknesses of digital peer support from the point of view of the user which is important.

Response: Thank you for your kind and helpful feedback.

Intro
1. With regards to the introduction, I think it is reasonably well written and provides many relevant references to ground the paper, however I do feel the authors could enhance the references in this section surrounding self harm and prevalence / risks / challenges in youth psychiatry, going beyond the peer support groups (eg. Is the prevalence higher among those in support groups compared to those not).

Response: thank you for suggesting this, however my co-authors and I would like to clarify that self-harm is not a focus of this paper as it instead covers young people’s mental health and wellbeing more generally. We do reference an example in the introduction of a peer support platform for people who self-harm, but this is just to highlight that a possible risk of peer support is that it can exacerbate difficulties in some cases.

I find some of the language in the intro rather repetitive. Several times the author makes the same point that apps can be helpful, but on the other hand it has downfalls. And that there is research but at the same time lacking in research. I think the author should consolidate these thoughts perhaps in a more complete paragraph that frames the arguments for, and then another paragraph with the downfalls and then make the case for why more research is needed, instead of doing this multiple times every few sentences. The authors should consider spending more time in the intro also covering a background on reflexive thematic analysis

Response: the introduction has now been edited to be organised into a brief introductory paragraph, then the arguments for peer support, the arguments against, and then the need for more research, as you have suggested. We hope that this now reads better. With regards to your suggestion of covering more background on reflexive thematic analysis in the introduction, we have now clarified on page 6 line 114-117 that this was the method used:

‘The current study aims to investigate the experience of young adults engaging with Tellmi, a UK-based digital mental health peer support smartphone app for young people, using qualitative interviews and reflexive thematic analysis.’

However, in keeping with usual convention, details of the method of analysis are contained within the methods section. In response to various reviewers comments, more detail regarding the thematic analysis has been added to the data analysis section within the methods.

Methods    
2. Which researcher did these interviews? Was it only 1 researcher who did them all?

Response: this has now been noted on page 9 line 193-194:

‘All interviews were conducted by a researcher (BC) with experience of qualitative research with young people on sensitive topics.’

3. The different mediums such as chat vs audio vs video is a bit unconventional and should be addressed as a potential limitation

Response: Whilst we acknowledge and appreciate that the different interview formats may elicit different types of data, and some things (e.g., non-verbal cues) may be missed in audio or chat interviews, we were eager to ensure the interviews were accessible for those who may not feel comfortable showing their face, or who may not be able to take part in an audio interview. We believe that this flexibility is a strength of our study as it allowed people to take part who may not otherwise have been able to if video interviews were offered. There is research to suggest that the format of the interview does not elicit data that is of worse quality - please see <https://www.ncbi.nlm.nih.gov/pmc/articles/PMC10401366/> and <https://journals.plos.org/plosone/article?id=10.1371/journal.pone.0263876>.

This has now been addressed within the paper on page 7 line 138-142:

‘Whilst the data gathered from a chat-based interview as opposed to audio/video may be different, it is important to ensure the interviews are accessible to all who wish to take part. It has been identified that interviews using this format are acceptable and do not elicit data of a worse quality (17,18).’

It has also been addressed in the strengths and limitations section on page 25 line 528-531:

‘Whilst we perceive a strength of this study to be the varying formats of interviews offered, it may still be that some users may have reservations about taking part in interview-based research. Anonymous surveys within the app may be appropriate for these individuals and should be considered for future research’

4. The authors speak a lot in the intro about youth and peer support groups however the actual methods report recruiting subjects aged 18-25. These creates some disconnect between the intro and the actual trial.

Response: thank you for the feedback, however, we have checked the content and references in the introduction and can confirm that it all relates to young adults or adults. This has been clarified in places, for example:

‘Research with people aged 18 and above suggests that digital peer support provides spaces to hear things about the experience of mental health that are typically ‘hidden’ within society’

‘Digital peer support has also been highlighted as a space to develop connections and friendships with people who can relate to each other’s experiences amongst participants aged 21+’

‘For example users of a self-harm forum were surveyed (mean age 21)’.

We hope this is now clearer.

5. It is not clear based on the methods whether or not the researchers were seeking users with any particular mental health condition or just any type of user on the platform, can the researchers please specify.

Response: Recruitment was open to any user on the platform with no inclusion criteria relative to specific mental health conditions. This has now been specified on page 8 line 158-160:

‘There were no exclusion criteria and no inclusion or exclusion criteria relative to specific mental health conditions, recruitment was open to all app users.’

6. What is the general usership of the Tellmi app? Are there hundreds, thousands, or millions or users? The authors note that there was a small population to recruit from and I wonder how that is the case if the app has a large usership

Response: It has now been clarified that Tellmi has roughly 6000 monthly users. However, as the study advert was programmed to appear as a post when scrolling within the primary feed, it may be that people did not access that part of the app or did not scroll far enough to see it. It may also be that people were interested did not meet inclusion criteria as some who expressed an interest in taking part were either below age 18 or above 25, for example. This information about recruitment barriers has been added to page 8 line 162-167:

‘We do not know how many app users saw the advert, but we do know that roughly 6,000 people use the app monthly and therefore may have seen it. As the advert was programmed to appear as a post when scrolling within the primary feed, users may not have accessed that part of the app or scrolled far enough to see it, which may have posed barriers to recruitment.’

7. Ethics approval: you said university faculty … of which university?

Response: the university has now been named ‘university of Bristol’

8. You state verbal consent but one participant was via chat function? How did they provide consent?

Response: Please note that all participants provided written informed consent in advance of the interview (as stated on page 8 line 176-177). This particular participant also gave consent in writing via the chat function. This has now been clarified on page 8-9 line 177-178:

‘The participant who participated via the chat function of Teams provided consent in writing during the chat.’

9. Is the distress protocol available in supplementary materials?

Response: thank you for this suggestion. It has now been included with the supplementary materials and this is referenced on page 9 line 181.

10. The authors say 11 ppl participated but list 12 people in the gender break down

Response: thank you for spotting this typo. It has now been corrected on page 11 line 222-223:

‘11 young people participated, they were aged 18-24 (mean 20.3) and identified as women (n=7), men (n=2), non-binary (n=1) and 1 preferred not to say’

Results
11. Line 211 grammatical confusion with the quote saying “they” and “my”

Response: this has now been edited to avoid confusion (page 13 line 271-273):

‘This sense of digital peer support as being friendly, welcoming, informal and familiar left most participants feeling like “I never have to be on my own for as long as I use the app” [P12].’

12. The authors should be consistent about punctuation  (the period) to either put the period after the [] or before the “ “

Response: Thank you for highlighting this, we’ve now edited to make it consistent so the period now comes after the [].

13. Currently the authors go back and forth with this formatting and sometimes do not put a . at all

Response: Thank you for highlighting this, we’ve now edited to make it consistent so the full stop now comes after the [].

14.  Line 318 the quote says “being left unread” but I think the participant actually probably said “on read” which is a popular term meaning someone read your message but did not reply. The transcription service may have mis heard this

Response: thank you for drawing our attention to this, but we can confirm that it is transcribed correctly.

15. Line 329 authors say “despite favorable comparisons” … I think it is not really suitable to compare this with clinical care as they are totally different things. There is a place for clinical care and place for digital support, but they are so far removed from one another it’s hard to truly compare them.

Response: this sentence is referring to the comparisons made by participants in regards to receiving support from a peer compared to a professional, so is grounded in the data. This has been edited to clarify this on page 19 line 390-392:

‘Despite the favourable comparisons between digital peer support and more clinical, professional support made by participants in theme one, there was a sense amongst some participants that there are perceived limits to what digital peer support can offer.’

16. Line 383 the authors use in consistent referencing style and should be fixed

Response: thank you, this has now been fixed.

17. Line 402 more inconsistency in the referencing style .. the authors could incorporate thoughts and text from those references and cite them in normal fashion

Response: thank you, this has now been fixed.
